# Assessing the Gun Violence Archive as an Epidemiologic Data Source for Community Firearm Violence in 4 US Cities

**DOI:** 10.1001/jamanetworkopen.2023.16545

**Published:** 2023-06-02

**Authors:** Ariana N. Gobaud, Christina A. Mehranbod, Elinore Kaufman, Jonathan Jay, Jessica H. Beard, Sara F. Jacoby, Charles C. Branas, Brady Bushover, Christopher N. Morrison

**Affiliations:** 1 Department of Epidemiology, Columbia University Mailman School of Public Health, New York, New York; 2 Division of Traumatology, Surgical Critical Care, and Emergency Surgery, University of Pennsylvania Perelman School of Medicine, Philadelphia; 3 Department of Community Health Sciences, Boston University School of Public Health, Boston, Massachusetts; 4 Division of Trauma Surgery and Surgical Critical Care, Temple University Lewis Katz School of Medicine, Philadelphia, Pennsylvania; 5 School of Nursing, University of Pennsylvania, Philadelphia; 6 Department of Epidemiology and Preventive Medicine, Monash University School of Public Health and Preventive Medicine, Melbourne, Australia

## Abstract

**Importance:**

Firearm injury is a major public health burden in the US, and yet there is no single, validated national data source to study community firearm violence, including firearm homicide and nonfatal shootings that result from interpersonal violence.

**Objective:**

To assess the validity of the Gun Violence Archive as a source of data on events of community firearm violence and to examine the characteristics of individuals injured in shootings.

**Design, Setting, and Participants:**

This cross-sectional observational study compared data on community firearm violence from the Gun Violence Archive with publicly available police department data, which were assumed to be the reference standard, between January 1, 2015, and December 31, 2020. Cities included in the study (Philadelphia, Pennsylvania; New York, New York; Chicago, Illinois; and Cincinnati, Ohio) had a population of greater than 300 000 people according to the 2020 US Census and had publicly available shooting data from the city police department. A large city was defined as having a population greater than or equal to 500 000 (ie, Philadelphia, New York City, and Chicago). Data analysis was performed in December 2022.

**Main Outcomes and Measures:**

Events of community firearm violence from the Gun Violence Archive were matched to police department shootings by date and location. The sensitivity and positive predictive value of the data were calculated (0.9-1.0, excellent; 0.8-0.9, good; 0.7-0.8, fair; 0.6-0.7, poor; and <0.6, failed).

**Results:**

A total of 26 679 and 32 588 shooting events were documented in the Gun Violence Archive and the police department databases, respectively, during the study period. The overall sensitivity of the Gun Violence Archive over the 6-year period was 81.1%, and the positive predictive value was 99.0%. The sensitivity steadily improved over time. Shootings involving multiple individuals and those involving women and children were less likely to be missing from the Gun Violence Archive, suggesting a systematic missingness.

**Conclusions and Relevance:**

These findings support the use of the Gun Violence Archive in large cities for research requiring its unique advantages (ie, spatial resolution, timeliness, and geographic coverage), albeit with caution regarding a more granular examination of epidemiology given its apparent bias toward shootings involving multiple persons and those involving women and children.

## Key Points

**Question:**

Is the Gun Violence Archive, an independent data collection group, a valid source of data for community firearm violence, including firearm homicide and nonfatal shootings that result from interpersonal violence?

**Findings:**

In this cross-sectional study of data from 4 US cities (Philadelphia, Pennsylvania; New York, New York; Chicago, Illinois; and Cincinnati, Ohio) from 2015 to 2020, the overall sensitivity of community firearm violence shooting events in the Gun Violence Archive was 81.1%.

**Meaning:**

The findings may support the use of the Gun Violence Archive in large cities for research requiring its unique advantages, albeit with caution regarding granular examination of epidemiology given systematic biases.

## Teaser

This cross-sectional study assesses the validity of the Gun Violence Archive as a source of data on events of community firearm violence and examines the characteristics of individuals injured by shootings in 4 US cities.

## Introduction

Community firearm violence, defined as firearm homicide and nonfatal shootings that result from interpersonal violence, is an epidemic in the US.^1^ Nearly 20 000 lives were lost in 2020 as a result of firearm homicides,^2^ representing a 30% increase from 2019. Although national homicide data are available through vital records culled by the National Violent Death Reporting System (NVDRS),^3^ there is currently no comprehensive national data source for community firearm violence. To effectively implement and evaluate prevention efforts, an accurate understanding of the totality of firearm injury epidemiology and changes over time remains a critical need for communities, practitioners, and policy makers alike.

Current research exploring firearm injury epidemiology and the impact of community firearm violence interventions relies on data from either the NVDRS or a variety of other less standardized and less consistently available administrative data. These include national and state trauma registries, hospital records, government and public health agencies, police department reports, and public media—all of which have important limitations for constructing accurate estimations of the incidence and prevalence of community firearm violence.^3,4,5,6,7^ Some of these sources are not available for research without extensive aggregation to protect individual identities and protected health or legal information. Publicly available data from government agencies are typically available for fatal injuries at the city or county level only.^5,8^ Data aggregated at these levels may be too spatially coarse to be informative when studying the context of firearm violence within specific and local communities.

Community firearm violence concentrates in small geographic areas.^9,10^ As such, local-level counts of community firearm violence are needed to inform targeted interventions. Research has shown police data to be more comprehensive than trauma registry data in terms of community firearm violence.^11,12,13^ A select number of police agencies collect data on both fatal and nonfatal shootings and make it available to the public. These data often include specific information on time and location of the shooting, demographics of the shooter and the individual injured, injuries, and relationships, enabling more detailed analyses. This granular information is often not provided in conventional crime reports, like through the Uniform Crime Reporting program,^5^ locally or nationally. Studies using local police department data are, therefore, limited to cities that provide a public registry of shooting incidents, yielding analyses that are not generalizable to the whole country.^14,15^

The Gun Violence Archive (GVA), an independent data collection group, has created a data set of firearm violence events from public records and public media across the US with location information similar to that found in police department data.^16^ In addition to providing needed information to the public, GVA offers the potential to expand the scope, flexibility, and timeliness of research on firearm injury. However, concerns about the methods and validity of data collection through GVA may limit its usefulness. GVA has allowed researchers to conduct analyses of fatal and nonfatal intentional interpersonal shootings on a national level,^17,18,19,20^ including studies examining the spike in firearm violence following COVID-19 containment policies. Some prior work has found that the annual counts of homicides in the GVA align closely with federal estimates,^21^ yet other work has found that the GVA data contain gaps.^22^ To our knowledge, no study has validated event-level counts of GVA incidents, although some studies^23,24^ have shown aggregate area-level counts of the GVA data to be correlated with data provided by police departments and the Centers for Disease Control and Prevention. Validation of GVA is vital so that researchers use the source appropriately and can account for any limitations.

To fill this gap, we aimed to validate community firearm violence shootings, which we refer to as *shootings* throughout this article, in the GVA at both the event and person levels. By validate, we mean to test the GVA’s ability to correctly identify shootings that occurred.^25^ First, we calculated the analytical sensitivity (ie, the probability of correctly diagnosing a case^25^) to capture the ability of the GVA to detect a shooting that it is intended to detect. Second, we calculated the positive predictive value (ie, the probability that a person with a positive test result is a true positive) to capture the proportion of shootings in the GVA that are truly shootings. In absence of a true reference standard for population-level firearm injury, we used police department data to validate GVA shootings. On the basis of prior findings that shooting incidence for GVA reports correlate highly with shooting incidence from police reports within geographic areas,^22,23^ we hypothesized that sensitivity and positive predictive values would be high.

## Methods

### Ethics

This cross-sectional study used only publicly available, anonymous data and was, therefore, not considered human participants research by the institutional review board of Columbia University; thus, informed consent was not needed, in accordance with 45 CFR §46. The study followed the Strengthening the Reporting of Observational Studies in Epidemiology (STROBE) reporting guideline.

### Data Sources

#### Gun Violence Archive

The GVA gathers daily information on firearm violence events from public records.^7^ According to their website, data are collected on shooting fatalities, as well as events “where a victim was injured by shooting or by a victim who was the subject of an armed robber or home invader.”^7^ They also collect data on incidents of “defensive gun use, home owners who stop a home invasion, store clerks who stop a robbery [and] individuals who stop an assault or rape with a gun.”^7^ Detailed methods on how the data are collected are not available; however, their website states that they “utilize automated queries, [and] manual research through over 7,500 sources from local and state police, media, data aggregates, government and other sources daily. Each event is verified by both researchers and a secondary validation process.”^7^ Existing fields in the data describe the date when the shooting event occurred, the street address with geographic coordinates of the shooting event, and the total number of individuals killed or injured in the incident. Each field also includes a link to a media report on the incident. These data are publicly available and searchable from fall 2013 forward.

#### Reference Standard

Cities included in the study had a population of greater than 300 000 people according to the 2020 US Census and had publicly available shooting data from the city police department. We chose 300 000 as the population cutoff because we hypothesized that larger city police departments are better resourced to collect these data. We used the search terms “city name” AND “police department shooting database” in Google to identify cities to be included. This procedure resulted in 4 cities: Philadelphia, Pennsylvania^26^; New York, New York^27^; Chicago, Illinois^28^; and Cincinnati, Ohio.^29^ We further categorized the cities as large (ie, having a population ≥500 000) and midsize (ie, having a population between 300 000 and 500 000). Philadelphia, New York City, and Chicago were all large cities, whereas Cincinnati was our 1 midsize city.

Detailed information regarding each city’s publicly available shooting data can be found in Table 1. Data used for this analysis included the date and location of the shooting along with the age, sex, race, and ethnicity of the individual injured in the shooting. The racial categories available differed by the data source. We categorized race and ethnicity as non-Hispanic Black, non-Hispanic White, and other (eg, American Indian/Alaska Native, Asian, Pacific Islander, and unknown) to accommodate the similarities and differences across data sources. Race and ethnicity were analyzed in this study to determine whether GVA and police reports of firearm violence differed systematically with respect to the race and ethnicity of the individual injured in the shooting.

**Table 1.** Summary Table of Data Sources

Data source	Database description	Variables	Date range
Gun Violence Archive^16^	The Gun Violence Archive is a database of events of gun violence and gun crime defined as shootings. Data are gathered from automated queries, manual research through over 7500 sources from local and state police, media, data aggregates, government and other sources daily. Each event is verified by both initial researchers and secondary validation processes. Links to each event are included in the incident report.	Available fields in the Gun Violence Archive data describe the date when the shooting occurred, the address (eg, 8600 Woodridge Road, Tampa, FL) with geographic coordinates of the shooting event, and the total number of individuals killed or injured in the incident. Each field also includes a link to a media report on the incident.	Fall 2013 to present
Philadelphia Police Department^26^	The Philadelphia Police Department database contains all people shot as a result of interpersonal violence in the city of Philadelphia, including individuals shot by police officers. Information on individuals injured in shootings is entered manually by examining investigative reports from detectives.	Available fields in the Philadelphia Police Department data describe the time and date when the shooting occurred, the block-level location (eg, 5100 Block Saul St) with geographic coordinates of the shooting event, and whether the shooting occurred inside or outside. Each row signifies a single individual shot, and information is provided on the demographic characteristics of that individual (sex, age, race, and Hispanic ethnicity) and whether the shooting was fatal or nonfatal.	January 2015 to present
New York Police Department^27^	The New York City Police Department database includes every individual injured in a shooting as a result of interpersonal violence in New York City. The data are manually extracted every year and reviewed by the Office of Management Analysis and Planning before being posted on the New York City Police Department website.	Available fields in the New York City Police Department data describe the time, date, and geographic coordinates of the shooting event. Each row signifies a single individual shot, and information related to suspect and victim demographics (sex, age, race) and whether the shooting was fatal or nonfatal are included.	January 2006 through end of previous calendar year
Chicago Police Department^28^	The Chicago Police Department database includes individual-level homicide and nonfatal shootings, not including shootings by police officers. The data set is refreshed daily, excluding the most recent complete day.	Available fields in the Chicago Police Department data describe the partially redacted block-level location where the shooting occurred (eg, 3000 Jackson Blvd) with geographic coordinates of the shooting event. Each row signifies a single individual shot, and information related to the individual’s demographics (sex, age, race) and whether the shooting was fatal or nonfatal are included.	Homicide data from January 1991 to present, and nonfatal shooting data from January 2010 to the present
Cincinnati Police Department^29^	The Cincinnati Police Department database includes confirmed shootings in the City of Cincinnati and is refreshed daily.	Available fields in the Cincinnati Police Department data describe the block-level location where the shooting occurred (eg, 6200 Joyce Ln) with geographic coordinates of the shooting event. Each row signifies a single individual shot, and information related to the individual’s demographics (sex, age, and race) and whether the shooting was fatal or nonfatal are included.	January 2008 to present

### Statistical Analysis

#### Event Level

We calculated event-level sensitivity and positive predictive value of the GVA data pooled over the 6-year period for all cities and for each city per year separately (values of 0.9-1.0 were considered excellent; 0.8-0.9, good; 0.7-0.8, fair; 0.6-0.7, poor; and <0.6, failed) (Table 2).^30^ To do this, we compared the count of shooting events from the GVA with that from the police department data. First, we aggregated events in each respective police department database that were likely part of the same event, following a method proposed by Beard and colleagues.^31,32^ Data for all firearm-injured individuals were ordered sequentially according to the date and time of the shooting. Each person shot was grouped with any other individuals shot within 1 hour and within 100 m (approximately 1 city block). Groups containing 2 or more firearm-injured individuals were part of the same event.

**Table 2.** Data Used to Calculate Sensitivity and Positive Predictive^,^ Value^a^^,^^b^

Data available from Gun Violence Archive	Data available from police department records, No. of cases		Total No. of cases
	Yes	No	
Yes	26 420 (true-positive)	259 (false-positive)	26 679
No	6168 (false-negative)	Not applicable (true-negative)	6168
Total	32 588	259	32 847

^a^ Sensitivity = true-positive / (true-positive + false-negative).

^b^ Positive predictive value = true-positive / (true-positive + false-positive).

For this analysis, we extracted the shooting events from the GVA and the police department databases from January 1, 2015, to December 31, 2020. This study period includes the spike in firearm violence that occurred after the start of the COVID-19 pandemic, as well as prior years in which US firearm violence was rising incrementally.^33^ We set the start date as January 1, 2015, because the GVA states on its website the number of sources they reference in their primary source list nearly doubled in 2015.

Variables common in both data sets and relevant to this analysis were the date and the location of the shooting. Using the police department data as complete, we matched GVA shooting events to the police department events occurring on the same date and were geolocated within 100 m.

#### Person Level

In addition to the event-level analyses, we conducted 2 person-level analyses to determine whether GVA and police reports of firearm violence differed systematically with respect to characteristics of individuals injured in shootings. First, we identified the characteristics of the individuals missing from the GVA after event-level matching, in all cities pooled over the 6-year period. Second, we conducted a multivariable logistic regression model incorporating characteristics of injured individuals, shooting characteristics, a fixed effect for year, and a random effect for city to identify independent factors associated with whether an individual injured in a shooting was missing from the GVA. We then removed the random effect and repeated this analysis for each city separately.

All event-level and person-level analyses were performed using R statistical software version 4.0.5 (R Project for Statistical Computing). Data analysis was performed in December 2022.

## Results

### Event Level

From January 1, 2015, to December 31, 2020, there were 26 679 shooting events in the GVA data and 32 588 shooting events in the police department data for Philadelphia, New York City, Chicago, and Cincinnati. The overall sensitivity of the GVA over the 6-year period was 81.1%, and the positive predictive value was 99.0%. The sensitivity of the GVA data increased over time, whereas the positive predictive value remained stable (Table 3). The sensitivity was lowest in 2016 (68.8%) and highest in 2020 (91.7%). This value varied by city.

**Table 3.** Event-Level Sensitivity and Positive Predictive Value of the Gun Violence Archive by Year and City, 2015-2020

Variable	Year					
	2015	2016	2017	2018	2019	2020
Sensitivity, %						
Overall	72.1	68.8	76.6	86.5	90.2	91.7
Philadelphia, Pennsylvania	47.3	50.3	49.2	93.4	96.6	99.5
New York, New York	55.3	51.9	59.2	58.2	82.1	84.8
Chicago, Illinois	92.2	80.9	94.2	94.9	93.5	95.7
Cincinnati, Ohio	60.5	52.0	55.9	60.1	63.2	50.2
Positive predictive value, %						
Overall	98.9	99.0	98.8	98.9	98.7	99.6
Philadelphia, Pennsylvania	99.4	99.5	99.8	99.7	99.8	100.0
New York, New York	99.2	97.0	96.5	97.1	98.8	99.5
Chicago, Illinois	99.9	100.0	100.0	100.0	99.3	99.9
Cincinnati, Ohio	88.8	88.7	88.1	85.2	87.5	92.2

### Person Level

During the study period, there were 29 791 individuals involved in shootings reported in both the police department and the GVA, of which 20 760 (69.7%) were nonfatal, 26 002 (87.3%) were involved in shootings with multiple injured individuals, 26 376 (88.5%) were male, 23 334 (78.3%) were individuals racialized as Black, and 25 005 (83.9%) were adults. The GVA was missing 9643 individuals involved in shootings, of which 6036 (62.6%) were nonfatal, 7913 (82.1%) involved single injured individuals, 8755 (90.8%) were male, 7742 (80.3%) were individuals racialized as Black, and 8608 (89.3%) were adults (Table 4). In multivariable logistic regression of factors associated with shooting-injured individuals missing from the GVA for all cities pooled over the 6 years (Table 5), female individuals were less likely than male individuals to be missing from the GVA (odds ratio, 0.81; 95% CI, 0.75-0.88), and children (aged <18 years) were less likely than adults to be missing (odds ratio, 0.81; 95% CI, 0.75-0.87).

**Table 4.** Descriptive Characteristics for Shootings in Both Police Department Database and the GVA Compared With Shootings Missing From the GVA, 2015-2020

Variable	Shootings, No. (%)	
	Police department and GVA (n = 29 791)	Missing from GVA (n = 9643)
Outcome		
Fatal	9031 (30.3)	3607 (37.4)
Nonfatal	20 760 (69.7)	6036 (62.6)
Individuals shot		
Single	3789 (12.7)	7913 (82.1)
Multiple	26 002 (87.3)	1730 (17.9)
Sex of individual shot		
Female	3415 (11.5)	888 (9.2)
Male	26 376 (88.5)	8755 (90.8)
Race and ethnicity of individual shot		
Black (non-Hispanic)	23 334 (78.3)	7742 (80.3)
White (non-Hispanic)	1727 (5.8)	653 (6.8)
Other^a^	4728 (15.9)	1248 (12.9)
Age of individual shot		
Child (<18 y)	4786 (16.1)	1035 (10.7)
Adult	25 005 (83.9)	8608 (89.3)

Abbreviation: GVA, Gun Violence Archive.

^a^ Other refers to American Indian/Alaska Native, Asian, Pacific Islander, and unknown.

**Table 5.** Multivariable Logistic Regression Results Showing Factors Associated With Individuals Injured in Shootings Missing From the Gun Violence Archive in Select Cities, 2015-2020

Variable	OR (95% CI)				
	Model 1: all cities all years	Model 1: Philadelphia, Pennsylvania	Model 2: New York, New York	Model 3: Chicago, Illinois	Model 4: Cincinnati, Ohio
Shooting characteristics					
Fatal	1.01 (0.95-1.07)	0.37 (0.32-0.43)^a^	6.83 (5.73-8.20)^a^	0.96 (0.88-1.05)	0.12 (0.08-0.16)^a^
Nonfatal	1 [Reference]	1 [Reference]	1 [Reference]	1 [Reference]	1 [Reference]
Sex of individuals shot					
Female	0.81 (0.75-0.88)^a^	0.66 (0.55-0.79)^a^	0.53 (0.45-0.64)^a^	1.11 (0.99-1.24)	0.61 (0.46-0.79)^a^
Male	1 [Reference]	1 [Reference]	1 [Reference]	1 [Reference]	1 [Reference]
Race and ethnicity of individuals shot					
Black (non-Hispanic)	1.01 (0.92-1.12)	1.06 (0.93-1.20)	1.21 (0.87-1.69)	0.76 (0.62-0.95)^a^	0.93 (0.70-1.24)
Other^b^	0.84 (0.75-0.95)^a^	0.92 (0.49-1.64)	1.06 (0.76-1.50)	0.61 (0.49-0.78)^a^	0.32 (0.13-0.74)^a^
White (non-Hispanic)	1 [Reference]	1 [Reference]	1 [Reference]	1 [Reference]	1 [Reference]
Age of individual shot					
Child	0.81 (0.75-0.87)^a^	0.61 (0.50-0.74)^a^	0.60 (0.49-0.73)^a^	0.89 (0.81-0.98)^a^	0.81 (0.61-1.09)
Adult	1 [Reference]	1 [Reference]	1 [Reference]	1 [Reference]	1 [Reference]

Abbreviation: OR, odds ratio.

^a^ Denotes statistical significance at α = .05.

^b^ Other refers to American Indian/Alaska Native, Asian, Pacific Islander, and unknown.

## Discussion

To our knowledge, this cross-sectional study is the first to validate the GVA at the shooting event level and over multiple cities and multiple years.^22,23^ Our analysis of the GVA identified nearly one-fifth fewer shooting events than the police department data over the 6-year period in Philadelphia, New York City, Chicago, and Cincinnati. We found good sensitivity of the GVA overall. Sensitivity steadily improved over time, reaching excellent levels in the 3 large cities by 2019 and poor sensitivity for the 1 midsized city. In addition, we found excellent positive predictive value. In this context, nearly all the shootings in the GVA over multiple cities and years were truly shootings. This is not surprising because each incident documented in the GVA is accompanied by some form of a media source. It is unlikely for a false positive, or an incident that was not a shooting incident, to be documented in the GVA. Despite the overall good sensitivity and nearly perfect positive predictive value, there may be specific causes for interpretive caution when extrapolating a more granular epidemiologic examination given the noted bias toward overrepresentation of shootings involving multiple individuals and those involving women and children.

Fatal shootings and shootings involving women and children were less likely to be missing from the GVA, suggesting a systematic bias related to how the data are collected and what kind of data are included. One potential explanation for this finding is that the biases we observed in the GVA data reflect the biases in media reporting on community firearm violence. Prior research^34^ investigating news media reporting on crime and violence has shown that homicides of women, children, and White individuals receive more news coverage than homicides of people from other demographic groups. In addition, news media have a long history of racializing violence coverage, underreporting crimes involving Black individuals who are injured and disproportionally covering stories involving Black crime suspects.^35,36^ Because GVA data rely heavily on media sources, this inherently impacts the completeness of the GVA and could introduce systematic biases in the data that are worthy of future study.

The results of our study also indicate that changes in sensitivity over time are of concern when using GVA data as a source to study trends in shootings. For example, many studies relied on the GVA to examine community firearm violence and the COVID-19 pandemic.^17,18,19,37^ There was no apparent decrease in the sensitivity of the GVA during the COVID-19 pandemic, at least for major cities. This is important because the pandemic was associated with a spike in community firearm violence, presumably without a commensurate increase in media reporting or GVA capacity. Future studies on midsize cities like Cincinnati and even smaller cities are required to understand this phenomenon further. The value of timely data from the GVA during the pandemic was high, given the time lag in official sources. However, studies relying on data from the GVA alone may have overestimated COVID-19–related spikes, since GVA sensitivity in the cities in this study was increasing before and during the pandemic.

### Limitations

We must interpret the results with some limitations in mind. First, there is no true reference standard for the incidence of community firearm violence that can be used as a comparator. Research conducted in Philadelphia found police data to be more comprehensive than trauma registry data for enumerating the incidence of community firearm violence.^12^ We made the assumption that this would be similarly true in the other 3 cities included in the analysis. However, in lieu of an alternate data source with the required granular information on community firearm violence, police department data were the best possible option. Second, we are unable to assess whether changes in sensitivity over time reflect improvements to the GVA method or changes in media coverage and other public data availability. We hypothesize the latter because the improvement in sensitivity varied across cities. The GVA’s inherent susceptibility to changes in media coverage is a matter of continued concern when using it as a data source. Specifically, public attention to crime is highly political and could influence the GVA and explain the apparent variability in their data. With regard to data availability, changes in sensitivity may be related to when police data became publicly available. For example, the Philadelphia Police Department Shooting Victims data became available mid-2016, which would align with the GVA’s increase in sensitivity. Third, we are unable to calculate the specificity or accuracy (ie, relative lack of error^25^) of the GVA because there is no way to capture true negatives, or when shootings do not occur. Fourth, our results have limited generalizability. We choose 4 cities according to population size and data availability. Although we found generally similar results, results may differ in important ways, particularly among other smaller cities in suburban and rural areas. It is possible the sensitivity of the GVA is stronger in smaller cities because the GVA is more likely to have complete data. However, a strength of our research is that we included 6 years of data. We can see a trend toward increasing the validity of the GVA over time. Fifth, we were not able to include assessment on the intent of each shooting event because of a lack of detailed information from both the GVA and police department data.

Data availability limits the study of firearm injury epidemiology in the US. Even when data are available, there is variability in the information provided, causing challenges in conducting research generalizable beyond a local or regional context. Having uniformity of variables across data sets would be useful to researchers interested in multicity studies of community firearm violence and would likely improve the accuracy of the GVA. Recognizing that the GVA is a small, independent data collection group, perhaps there are ways to collaborate with local police departments or the Centers for Disease Control and Prevention with a shared mission to provide comprehensive data for community firearm violence research. Specifically, the field would benefit from a data source that includes data on all types of firearm injury and includes geographic data. In lieu of that, researchers should proceed with caution using the GVA data. We recommend against using the GVA for any time trends analyses because of the low sensitivity in the earlier years (particularly in smaller cities) and considering the impacts of the data’s systematic biases on any found results.

## Conclusions

The granularity of our event-level and person-level validation along with the description of time trends extends prior research that has attempted to validate the GVA with aggregate area-level counts of data. Our findings generally support the use of GVA for research that requires its unique advantages (ie, spatial resolution, timeliness, and geographic coverage), albeit with caution using GVA data from earlier years and examining trends over time. Moreover, studies using GVA data should acknowledge that their results may be biased as a result of systematic missingness. Future research should assess the validity of the GVA in other cities to see whether the GVA can be considered a valid national source of community firearm violence. In particular, the validity of the GVA in cities that do not have available police department data needs to be examined before the GVA could be considered a national valid data source of community firearm violence.

## Notes

Supplement.
Data Sharing Statement
