# Supplementary material for: Assessing the Gun Violence Archive as an Epidemiologic Data Source for Community Firearm Violence in 4 US Cities
Source: JAMA Netw Open. 2023 Jun 2;6(6):e2316545. doi: 10.1001/jamanetworkopen.2023.16545 (PMC10238941; doi:10.1001/jamanetworkopen.2023.16545)
Supplement: Supplement. — Data Sharing Statement [file jamanetwopen-e2316545-s001.pdf]

Data Sharing Statement

Gobaud. Assessing the Gun Violence Archive as an Epidemiologic Data Source for Community Firearm Violence in 4 US Cities. *JAMA Netw Open*. Published June 02, 2023. doi:10.1001/jamanetworkopen.2023.16545

Data

Data available: All data used for this study are publicly available. The dataset used in this study may be made available upon reasonable request to the corresponding author.
